# ^18^ F-FDG PET-CT Evaluation of Primary Adrenal Ewing Sarcoma with Venous Thrombosis: An Unusual Presentation

**DOI:** 10.1055/s-0042-1757251

**Published:** 2022-09-09

**Authors:** Debdip Roy, Melvika Pereira, Divya Shivdasani, Natasha Singh

**Affiliations:** 1 Department of Nuclear Medicine and Molecular Imaging, P. D. Hinduja National Hospital and MRC, Mahim, Mumbai, Maharashtra, India

**Keywords:** adrenal, Ewing sarcoma, FDG PET-CT, IVC thrombus

## Abstract

Ewing sarcoma (EWS) is primarily an osseous malignancy of childhood and young adults. Extraskeletal occurrence is less frequent and primary adrenal involvement is an even rare presentation. We present such a case of a 7-year-old boy diagnosed with adrenal EWS with associated venous thrombosis and pulmonary embolism detected on ^18^ F-fluorodeoxyglucose positron emission tomography-computed tomography scan.

## Introduction

Ewing sarcoma (EWS) is a round cell tumor primarily affecting skeletal system, with a higher incidence in children and young adults after osteogenic sarcoma among skeletal malignancies. Although rare, primary extraosseous EWS has been reported involving chest wall, paravertebral and retroperitoneal region, and even rarer occurrence of organ involvement (lungs, gastrointestinal, prostate, others). 1 2 Primary adrenal EWS is unusual in pediatric population with very few cases reported till date. We present such a case of primary adrenal EWS in a 7-year-old boy with tumor thrombus involving renal vein and inferior vena cava (IVC) with pulmonary thromboembolism detected on positron emission tomography-computed tomography (PET-CT). The case emphasizes on considering EWS as a differential diagnosis in aggressive presentation of adrenal mass lesions in pediatric population and role of ^18^ F-fluorodeoxyglucose positron emission tomography-computed tomography ( ^18^ F-FDG PET-CT) scan in assessing disease burden that can guide diagnosis and early management.

## Case Presentation

**Fig. 1:**
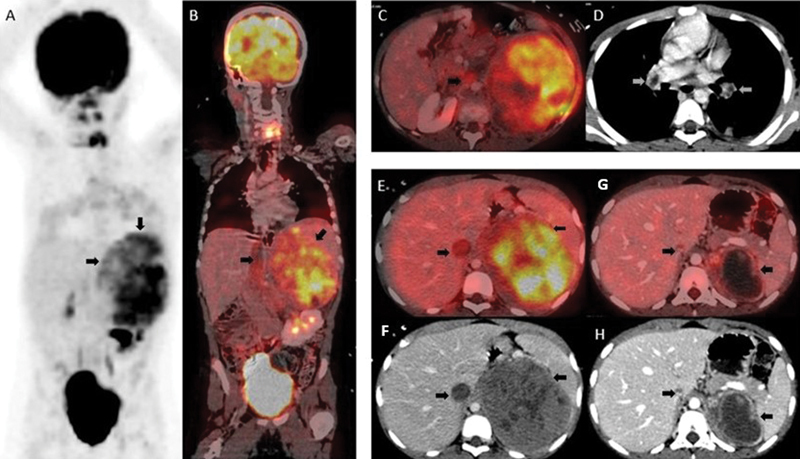
Baseline PET-CT MIP ( **A** ), fused PET-CT coronal ( **B** ), transaxial ( **E** ), CECT axial ( **F** ) images show FDG avid large, heterogeneously enhancing left adrenal mass with hypodense-necrotic areas within, associated FDG avid tumor thrombus in left suprarenal vein, both renal veins, IVC reaching upto right atrium ( **C** ). Bilateral pulmonary thromboembolism was detected ( **D** ). Post chemotherapy PET-CT ( **G** ), CECT ( **H** ) images show significant decrease in size and FDG uptake of left adrenal lesion and IVC thrombus.

A 7-year-old boy, presenting with complains of paroxysmal seizure, tachycardia, hypertension, and respiratory distress and a palpable abdominal mass in left hypochondrium, was admitted at our institution. Magnetic resonance imaging (MRI) abdomen done at another center suggested a large left suprarenal mass lesion raising possibility of neuroblastic tumor. Laboratory investigation revealed normal plasma cortisol, plasma-free metanephrine and normetanephrine, and urinary metanephrine levels. Patient was referred to our department for ^18^ F-FDG PET-CT scan to further characterize the lesion and evaluate the disease extent. The scan taken after 1 hour of 4 mCi of ^18^ F-FDG injection. PET-CT scan showed high-grade FDG avid large, lobulated, heterogeneously enhancing left adrenal mass lesion (maximum standardized uptake value: 9.2) with hypodense necrotic areas within and associated thrombus in left suprarenal vein, extending in both renal veins, and IVC reaching upto right atrium, with heterogeneous components within showing mild FDG uptake suggesting tumor thrombus. In addition, bilateral pulmonary thromboembolism was detected ( Fig. 1A–F ). No lymphadenopathy or distant metastasis was seen. Based on the clinical and PET-CT findings, possibility of adrenocortical tumor was considered over neuroblastoma. However, CT-guided biopsy evaluation of left adrenal mass revealed EWS, immunohistochemical (IHC) profile showing intense expression of CD99, with K _i_ -67 index of 35%. After multidisciplinary discussion, the patient was treated with chemotherapy including vincristine, ifosfamide, doxorubicin, and etoposide, showing good symptomatic response. Treatment response PET-CT scan was performed after three cycles of chemotherapy that showed significant decrease in size and FDG uptake of primary left adrenal neoplastic lesion, and decrease in extent of IVC thrombus with resolution of thrombus in renal and suprarenal vein, and pulmonary thromboembolism ( Fig. 1G, H ). Patient is doing clinically well on chemotherapy.

## Discussion

EWS is the second most common skeletal malignancy affecting adolescents after osteogenic sarcoma. 3 Extraskeletal EWS is less frequently encountered and involves soft tissue and rarely even organ involvement. Primary involvement of adrenal gland in EWS being extremely rare is not considered among the usual differential diagnosis of adrenal mass in children. 2 Rather, based on the age group and site of involvement in our case neuroblastoma and possibly adrenocortical carcinoma were our major differentials. Detection is commonly done on CT scan or MRI, whereas in our case adrenal mass was characterized on whole body FDG PET-CECT scan. These modalities help in differentiating benign from neoplastic adrenal lesion. Adrenal EWS lesions have been reported as mass lesion with heterogeneous enhancement, areas of necrosis or internal foci of calcification and being locally aggressive in nature may involving adjacent vessels. 4 5 6 Our case showed similar features of large lobulated heterogeneously enhancing adrenal mass with central areas of necrosis with tumor thrombus extending into adjacent renal vein and IVC. Since adrenal involvement formed major bulk of the lesion on PET-CT scan, possibility of adrenal metastasis was ruled out. There are no reports yet on detection of primary EWS on FDG PET, and our case shows that lesions demonstrate FDG avidity considering their aggressive nature. Although there are no specific imaging findings on PET-CT pertaining to primary adrenal EWS, FDG PET-CT scan can help in staging by determining the disease burden and thus guiding management. Additionally in our case due to whole body imaging, bilateral pulmonary thromboembolism was detected. Histopathology is the gold standard to determine this rare diagnosis, and EWS pathological features include round blue cell tumor positive for pancytokeratin, membranous CD99 on IHC, and reverse-transcription polymerase chain reaction positive for EWSR1-FL1 gene that are highly sensitive. 7 8 Biopsy performed in our patient showed similar IHC characteristics. Treatment strategy includes surgery and chemotherapy both. Our patient received chemotherapy demonstrating a good response on subsequent posttreatment PET-CT. So, we see another benefit of FDG PET in assessment of treatment response in EWS. Our case thus highlights the rarity of the diagnosis of primary adrenal EWS with locally aggressive features detected on staging FDG PET-CT scan, and the importance of including EWS as one of the differential diagnoses of adrenal masses. Also, we must note that since these lesions are FDG avid, FDG PET-CT is a useful modality in assessing metastatic burden and treatment response, both of which guide management decisions.
